# Tailored Systemic Therapy for Colorectal Cancer Liver Metastases

**DOI:** 10.3390/ijms222111780

**Published:** 2021-10-29

**Authors:** Carolin Czauderna, Kim Luley, Nikolas von Bubnoff, Jens U. Marquardt

**Affiliations:** 1 Department of Medicine I, University Medical Center Schleswig-Holstein—Campus Lübeck, 23558 Lübeck, Germany; carolin.czauderna@uni-luebeck.de; 2 Department of Hemato-Oncology, University Medical Center Schleswig-Holstein—Campus Lübeck, 23558 Lübeck, Germany; Kim.Luley@uksh.de (K.L.); Nikolas.vonBubnoff@uksh.de (N.v.B.)

**Keywords:** colorectal cancer, molecular biomarkers, prognosis, prediction

## Abstract

Liver metastases are the most common site of metastatic spread in colorectal cancer. Current treatment approaches involve effective systemic therapies in combination with surgical and/or interventional strategies. Multimodal strategies greatly improved clinical outcomes of patients with metastatic colorectal cancer over the last decades. Identification of predictive and prognostic biomarkers helped to comprehensively refine individual targeted treatment approaches and resulted in median overall survival rates of 30 months or longer. Current guidelines, thus, recommend treatment selection according to patients’ performance status, tumor localization and stage as well as the tumor’s molecular and genetic status. Here, we outline the latest developments in molecular decision-making for patients with upfront resectable, potentially or initially unresectable and non/never-resectable colorectal cancer liver metastases.

## 1. Introduction

Colorectal cancer (CRC) is the third most common cancer and leading cause of cancer related death worldwide [1]. When diagnosed in late stages of the disease, patient prognosis remains poor [2]. Prevention, such as precautionary colonoscopy, remains the best strategy against CRC. Various further methods have been proposed with an increased interest in non-invasive biomarkers [3]. However, clinical outcomes of patients with metastatic disease (mCRC) have also improved significantly over the last decades due to identification of prognostic and predictive molecular biomarkers and subsequent individual refinement of treatment strategies [4,5]. The significant improvement in overall survival in patients with mCRC is based on an increasing number of patients that are treated in specialist cancer centers by multidisciplinary teams. Most importantly, application of multimodal treatment approaches, including effective and biomarker-based systemic therapies as well as resection and local ablation of metastases, is now considered standard of care for therapy of mCRC [5]. Therapeutic decision-making for patients with mCRC is mainly based on (i) patients’ performance status (PS), (ii) the extent and localization of disease and (iii) molecular profiles [6,7]. Combined chemotherapy with a fluoropyrimidine backbone (fluoropyrimidine/leucovorin (5-FU/LV) or capecitabine) together with irinotecan (FOLFIRI) or oxaliplatin (FOLFOX), or a combination of both (FOLFOXIRI), is the standard systemic therapy. First-line regimens are generally combined with targeted approaches including monoclonal antibodies directed towards vascular endothelial growth factor (VEGF), i.e., bevacizumab, or towards the epidermal growth factor receptor (EGFR) with panitumumab and cetuximab. Recently, immune checkpoint inhibitors (ICI) also received FDA approval for first-line treatment in patients with mCRC that harbor microsatellite instability. For further lines of therapy, other anti-angiogenic therapies as well as molecularly guided therapeutic strategies have been identified, especially for BRAF-mutated mCRC [8]. Several guidelines have been developed based on results of randomised phase III clinical trials, and provide evidence-based recommendations to assist decision-making in this rapidly evolving treatment landscape [5,7,9].

This review outlines key aspects of decision-making for patients with colorectal liver metastases (CLM), focusing on predictive and prognostic molecular biomarkers. A literature research of the last ten years for mCRC, using the PubMed platform with keywords such as ‘colorectal molecular biomarkers’, ‘colorectal liver metastasis’, ‘colorectal systemic therapy’ and ‘colorectal cancer treatment’, was performed. Therapeutic algorithms and treatment regimens described in this review will be classified and delineated following the European Society of Medical Oncology (ESMO) guidelines into (i) resectable (i.e., ESMO group 0) or (ii) potentially resectable (i.e., ESMO group 1) and (iii) non/never-resectable CLM (i.e., ESMO group 2 and 3) [6]. 

## 2. General Treatment Considerations Based on Predictive and Prognostic Molecular Biomarkers 

In cases of metastatic disease, molecular profiling of CRC has become a pre-requisite for optimal therapeutic decision-making and is based on a high level of evidence. 

The most commonly affected molecular alterations that should be explored are Kirsten rat sarcoma (KRAS) and Neuroblastoma rat sarcoma (NRAS) mutations occurring in 45% of CRC, BRAF mutations in 9%, HER2 amplification in 4%, and mismatch repair deficiency (MMRd)/microsatellite instability (MSI-H) in approximately 5%, along with many others that occur at a frequency of less than 5%. Upfront testing is broadly established for mutations in genes of RAS and BRAF as well as MMRd/MSI-H due to their high predictive and/or prognostic value [5]. 

### 2.1. RAS 

RAS mutations are crucial predictive biomarkers for therapeutic choice of EGFR antibody therapy in mCRC. Results of multiple controlled randomised trials showed that patients with mCRC with wildtype RAS show a significantly improved treatment response when treated with chemotherapy in combination with monoclonal antibody therapy targeting EGFR, compared to those that harbored any RAS mutation [10,11]. Results have been confirmed by several systemic reviews and meta-analyses including 5948 patients [12]. Importantly, BRAF mutations of codon V600 and RAS mutations are mutually exclusive. 

### 2.2. BRAF 

BRAF mutations, mainly the BRAF V600E mutation, have a high negative prognostic impact [13]. In comparison to other more infrequent BRAF mutations of codons D594 and G596, BRAF V600E mutations have been detected more frequently in right-sided primary tumors with peritoneal metastases [14]. Furthermore, mCRC with BRAF V600 mutations, albeit not associated with germline MMR mutations, more frequently present with MSI with sporadic defects in mismatch repair genes [15]. 

### 2.3. MMRd/MSI-H 

MMRd/MSI-H status is associated with a high tumor-mutational burden and, thus, relevant as positive predictor for immune-checkpoint inhibition (ICI) in first- and second-line therapy of mCRC [16,17,18]. 

In cases of refractory disease, further molecular markers, including HER-2 and PIK3CA among others, can be explored to identify additional targeted approaches [19].

Several studies have further investigated predictive value of non-invasive molecular biomarkers, i.e., liquid biopsies such as circulating long-coding RNAs or microRNAs for CRC. Meta-analyses and systemic-reviews conclude, that they have high diagnostic and prognostic value for patients with CRC [20,21,22]. However, no RNA-based biomarkers have yet entered routine clinical practice. Genetic heterogeneity of both, patient and CRC, might hamper translational breakthrough and future well-designed and standardized studies compromising large patient cohorts including high quality controls will be needed to promote this field of interest [23].

Established in clinical routine are further genetic tests that also predict sensitivity towards and toxicity of classical chemotherapies: Dihydropyrimidine dehydrogenase (DPD) is crucial for the metabolic catabolism of 5-FU and capecitabine. Deficiencies in DPD are associated with severe drug-related toxicities [24]. UDP glucuronosyltransferase 1 family, polypeptide A1 (UGT1A1), is an enzyme important for glucuronidation processes, i.e., glucuronidation of SN-38, the active metabolite of irinotecan [25]. Certain polymorphisms of UGT1A1 are predictive for irinotecan-related side effects, such as diarrhea, neutropenia and vomiting. DPD genetic testing is an option prior to therapy and UGT1A1 genetic testing should be considered, especially in patients who experienced severe toxicities and/or who have known bilirubin-conjugation disorders [5,7].

## 3. Resectable Liver Metastases 

Resection of CLM is considered the mainstay and, potentially, only curative treatment approach for patients with mCRC [26]. Multimodal (neo)adjuvant or perioperative treatment approaches combining surgery and systemic therapies have been investigated. In this context, perioperative treatment refers to chemotherapy application before and after surgery.

Clinical evidence for the efficacy of neoadjuvant treatments is low for patients with clearly resectable CLM. Few randomised controlled trials have been performed. They showed that therapy can be safely applied in this context, but did not demonstrate an advantage in overall survival (OS) in this patient population [27,28]. Similarly, the benefit of adjuvant/additive chemotherapy after resection of metastatic liver lesions has not been proven with sufficient statistical power [7,29]. A landmark study for perioperative treatments for patients with clearly resectable liver metastases was the EORTC-40983 trial [30]. The randomised, controlled, phase III trial investigated perioperative FOLFOX4 chemotherapy and surgery versus surgery alone for resectable CLM regardless of mutational status. Updated long-term analysis for patients randomly assigned to the treatment arms showed that median progression-free survival (PFS) did not differ significantly between perioperative chemotherapy (*n* = 182) and surgery alone (*n* = 182): 20.0 months versus 12.5 months, hazard ratio (HR) 0.81, 95% confidence interval (CI) 0.64–1.02, *p* = 0.068. Furthermore, no difference in OS was detected by addition of chemotherapy [30]. The New EPOC phase III trial investigated the addition of cetuximab to perioperative chemotherapy in KRAS wild-type (codons 12, 13, and 61), resectable or suboptimally resectable CLM (*n* = 257). Results of long-term analyses have been recently published and concluded that the median PFS did not differ significantly between chemotherapy plus cetuximab and chemotherapy: 15.5 months versus 22.2 months; HR 1.17, 95% CI, 0.87–1.56, *p* = 0.304. Median OS was significantly lower with chemotherapy plus cetuximab compared to chemotherapy alone: 55.4 months vs. 81.0 months; HR 1.45, 95% CI, 1.02–2.05, *p* = 0.036 [31,32]. Thus, perioperative treatment with chemotherapy plus cetuximab is not recommended in patients with clearly resectable CLM. Notably, the cited studies mainly included patients with good prognostic factors (i.e., 1–4 resectable CLM of mainly metachronous disease; KRAS- wildtype (wt) tumors). Systemic reviews and meta-analyses on clinical significance and prognostic relevance of molecular subtypes for patients with resectable CLM revealed that BRAF and KRAS mutations consistently confer poor clinical outcomes and insufficient responses in patients undergoing hepatic resection [33,34,35,36]. Interestingly, investigations on surgical resection margins revealed that presence of any RAS mutation was associated with a positive resection margin and adverse survival of the patients [37]. A different study consistently showed that non-anatomical, tissue-sparing hepatectomy was associated with poor disease free-survival in patients with KRAS-mutated tumors in comparison to anatomical hepatectomy [38]. Thus, it is conceivable that a wider surgical resection margin as well as anatomical resection might be beneficial in these patients. Taken together, results of available studies clearly highlight the clinical need for further characterization of the impact of mutational status in the context of resectable CLM and might guide treatment decisions for surgical procedures. 

## 4. Potentially Resectable or Initially Unresectable Liver Metastases 

Although the EPOC and new EPOC trials could not demonstrate a benefit of perioperative chemotherapy in patients with a favourable prognosis, guidelines suggest preoperative treatment in cases of unfavourable disease such as synchronic disease and/or in patients with initially sub-optimal, only potentially, or even unresectable CLM in order to improve subsequent resectability [5,7]. Recommendations are based on a few phase II and III clinical trials that investigated this strategy as a primary endpoint. Further data can be extrapolated from phase III trials that evaluated resectability in patients with mCRC as secondary endpoints or in subgroup analyses.

For patients with KRAS exon 2-wt and technically unresectable and/or ≥5 CLM, efficacy of FOLFOX and cetuximab (arm A) and FOLFIRI and cetuximab (arm B) was evaluated in the CELIM trial. Response rates of 68% in patients treated with FOLFOX and 57% in those treated with FOLFIRI were reached with one-third of R0 resections across both treatment arms [39]. Patients who achieved R0 resection had better median OS, at 53.9 months, compared to those who did not (21.9 months) [40]. Similar results were obtained for panitumumab in combination with either FOLFOX6 or FOLFIRI in patients with KRAS-wt with multiple or non-resectable liver metastases in the PLANET study [41]. However, in both mentioned studies, both treatment arms included targeted therapy. Thus, the design allowed for revealing the impact of different chemotherapies, rather than the relevance of intensified systemic therapy in this setting. A randomised, controlled phase II trial by Ye et al. addressed this question and compared resection rates and survival of KRAS-wt patients treated with either chemotherapy alone or in combination with cetuximab. The primary endpoint was the rate treatment conversions in CLM (*n* = 138). Cetuximab combined with chemotherapy resulted in significantly higher R0 resection rates of 25.7% versus 7.4% (*p* < 0.01) as well as in improved response rates and survival compared with chemotherapy alone [42]. Data of the recently published phase II VOLFI trial confirm these results. The study evaluated the activity and safety of mFOLFOXIRI-panitumumab vs. FOLFOXIRI alone in ECOG 0–1, primarily non-resectable mCRC patients [43]. In this study, two cohorts were analysed, cohort 1 with non/never-resectable mCRC and cohort 2 with patients considered for secondary resection of metastatic lesions with a primary endpoint of overall response rates (ORR). For all patients results showed a better ORR in the panitumumab arm vs. FOLFOXIRI alone (87.3% v 60.6%; odds ratio, 4.469; 95% CI, 1.61 to 12.38; *p* = 0.004). PFS was similar in both treatment arms, whereas OS showed a trend in favor of the panitumumab-containing arm (hazard ratio for death, 0.67; 95% CI, 0.41 to 1.11; *p* = 0.12). Secondary resection rates of metastases were also significantly higher with 21 (33%) of 63 patients (cohort 1, *n* = 6; cohort 2, *n* = 15) in the panitumumab arm compared with four (12.1%) of 33 patients in the control arm (OR, 3.63; 95% CI, 1.13 to 11.67; *p* = 0.02) [43]. Therefore, the addition of targeted therapy to chemotherapy might yield a higher efficiency and improve secondary resectability of CLM.

Intensified chemotherapy in the context of treatment conversion was recently addressed in the ATOM trial. The study performed a head-to-head comparison of bevacizumab versus cetuximab for initially unresectable CLM in KRAS-wt patients (*n* = 122). PFS, the primary endpoint of this phase II trial, did not differ significantly between patients in the bevacizumab-containing and cetuximab-containing arms with 11.5 and 14.8 months (hazard ratio 0.803; *p* = 0.33), respectively. Although response rates were higher in the cetuximab-containing arm (84.7% versus 68.4%), resection rates were similar across both treatment arms (49.2% and 56.1%) [44].

Further studies compared the impact of triplet chemotherapy versus doublet regimens. The METHEP trial was one of the first trials that randomised 125 patients to either standard (FOLFIRI/FOLFOX4) or intensified chemotherapy (FOLFIRI-HD/FOLFOX7/FOLFIRINOX) in potentially resectable or unresectable CLM. Based on the results of this trial it can be concluded that FOLFIRINOX offers a better conversion rate (67%) [45]. The randomised, controlled phase II (OLIVIA) trial evaluated the efficacy of bevacizumab with either mFOLFOX or FOLFOXIRI in patients with initially unresectable CLM regardless of mutational status. Non-resectability was defined as >1 of the following criteria: no possibility of upfront R0/R1 resection of all lesions, <30% residual liver volume after resection and metastases in contact with major vessels of the remnant liver. The ORRs were 81% with FOLFOXIRI-bevacizumab and 62% with mFOLFOX6-bevacizumab. Concurrently, overall resection rates were 61% with FOLFOXIRI-bevacizumab and 49% with mFOLFOX6-bevacizumab, resulting in prolonged PFS (18.6 months versus 11.5 months) [46].

The relevance of the KRAS status was investigated in the randomised, controlled Prodige 14-ACCORD 21 (METHEP-2) phase II trial. This multicenter trial compared the efficacy of targeted therapy with doublet chemotherapy versus targeted therapy with triplet chemotherapy for patients with initially unresectable CLM. Cetuximab was applied in KRAS-wt and bevacizumab in KRAS-mutated patients. Chemotherapy with FOLFIRINOX in combination with a target therapy resulted in significantly higher resection rates (56.9% versus 45.2%) and OS, while severe toxicity rates were similar between treatment arms. Resection rates were also similar for patients treated with bevacizumab (RAS-mutant) and cetuximab (RAS-wt) (44.7% vs. 55.6%; *p* = 0.087) [47]. Further studies investigated the impact of loco-regional therapies such as transarterial chemoembolization (TACE) or selective internal radiotherapy (SIRT) for treatment of CLM. The most robust data exist for SIRT application in the treatment of potentially resectable or initially unresectable liver metastases. The phase III SIRFLOX trial studied secondary resection of CLM with the addition of SIRT using yttrium-90 resin microspheres to FOLFOX-based chemotherapy versus FOLFOX alone. Primary endpoint was technical resectability of CLM. 472 patients were randomised without significant baseline differences between both treatment arms. Significantly more patients were resectable in the SIRT arm versus the control arm (38.1% vs. 28.9%; *p* < 0.001) [48]. Adding SIRT to chemotherapy therefore may improve the resectability of initially unresectable CLM. Clinical trials on TACE for CLM are limited and restricted to palliative care/unresectable CLM only. Results of phase I and II studies showed that TACE can be safely applied in combination with chemotherapy, with promising tumor responses [49,50,51,52].

Taken together, available data suggest that intensified systemic treatments achieve high response rates and resection rates of potentially or initially unresectable CLM and should be rigorously applied in initially unresectable patients (Table 1). Addition of loco-regional therapies to chemotherapy might improve down-staging of CLM, but need to be further investigated in randomised controlled trials. Overall, studies demonstrate improved long-term outcomes for patients responding to conversion therapy and undergoing secondary resection. Re-evaulation for treatment response and resectability should be performed early and every two months, in order to prevent overtreatment of secondarily resectable patients [53].

## 5. Non-Resectable Liver Metastases

For patients with mCRC without or unlikely chances of reaching resectability, systemic therapies are the standard-of-care considering tumor-, patient- and treatment-related factors [5,6,7]. In order to decide on a therapy regimen, patients are commonly subdivided into those with urgent need of intensive treatment due to severe symptoms or organ failure (ESMO group 2) with the aim of rapid tumor remission, and those with oligosymptomatic disease with the aim of disease control (ESMO group 3) [6].

### 5.1. First-Line Regimens

Both anti-VEGF- and anti-EGFR-directed therapies combined with chemotherapy regimens represent efficient and well-established treatment options for patients with mCRC in the first line [8]. The addition of biologicals to chemotherapy has been studied in several phase III trials and resulted in prolongation of PFS and favorable OS [54].

For decision-making for either anti-VEGF or anti-EGFR therapy, both the mutational status (i.e., resistance to anti-EGFR treatment in RAS-mutant mCRC), and the tumor localization have to be appreciated. Left-sided tumors derive from a different embryologic origin than right-sided tumors. Hindgut and midgut are biologically different tissues, with different physiological functions as well as differences in epigenetic alterations, i.e., methylation patterns, and other oncogenic changes. A pooled analysis of six randomised trials (FIRE3, CALGB 80405, PEAK, CRYSTAL, PRIME, and 20050181) investigated the prognostic and predictive impact of localization of primary tumor in patients with non-resectable RAS-wt mCRC [55]. Results confirm poor prognosis for patients with right-sided tumors compared to those with left-sided tumors in terms of mOS, mPFS, and ORR. In addition, while significant benefit for anti-EGFR combination therapy could be recognised in patients with left-sided tumors, benefit for right-sided tumors was less pronounced. Patients with right-sided RAS-wt tumors likely benefit more from chemotherapy plus bevacizumab compared to cetuximab. Therefore, the side of tumor manifestation needs to be taken into account for the choice of treatment regimen.

For left-sided mCRC, the mutational status of KRAS needs to be tested before treatment is started, since anti-EGFR treatments with cetuximab or panitumumab have been shown to be mainly effective in KRAS-wt patients [54,56]. Data on head-to-head comparison of anti-VEGF and anti-EGFR treatments in a first-line setting are provided by three randomised trials (FIRE-3, CALGB/SWOG 80405, and PEAK). The phase III FIRE-3 study compared the efficacy of FOLFIRI-cetuximab versus FOLFIRI-bevacizumab in patients with KRAS-wt tumors. The study failed to show significant differences in ORR and PFS. However, the secondary endpoint OS was significantly prolonged for combination with cetuximab (28.7 months compared to 25 months) [57]. The phase II PEAK trial consistently reported an improvement in median OS of 41.3 versus 28.9 months (HR, 0.63; 95% CI, 0.39 to 1.02; *p* = 0.058) for FOLFOX-panitumumab compared to FOLFOX-bevacizumab in KRAS exon 2 wt patients [58]. On the other hand, the largest head-to-head trial with 2334 randomised patients (CALGB/SWOG 80405 trial) compared FOLFOX with FOLFIRI (by investigator’s discretion) in combination with cetuximab or bevacizumab, and could not confirm these results and observed no significant difference across treatment arms [59]. Taken together, evidence for choosing an anti-EGFR over an anti-VEGF treatment in first-line settings in patients with mCRC and KRAS-wt mCRC indicates a benefit for anti-EGFR in left-sided tumors, but the data are still inconclusive.

Accumulating evidence established BRAF mutations as negative predictive biomarkers for EGFR antibody therapy. Two meta-analyses confirmed that efficacy of anti-EGFR treatment is higher in patients with RAS-wt/BRAF-wt tumors compared to those with RAS-wt/BRAF-mutant tumors [60,61]. Importantly, the ongoing phase II FIRE-4.5 trail (EudraCT Number: 2015-004849-11) might reveal new findings in this clinical scenario. The study investigates FOLFOXIRI plus cetuximab versus FOLFOXIRI plus bevacizumab as first-line treatment of BRAF-mutated mCRC with the primary endpoint of ORR. Current guidelines suggest intensive treatment regimens for this molecular subgroup including triplet chemotherapy (FOLFOXIRI plus bevacizumab) [5,7,9]. Indeed, several randomised trials demonstrated greater efficacy for triplet combination therapy [46,62]. A subgroup analysis of the TRIBE study confirmed the benefit of intensive chemotherapy in BRAF-mutated mCRC; however, since the subgroup was small (*n* = 28), results need to be interpreted with caution. Results from the phase II VOLFI trial also supported high ORR resulting from triplet therapy plus panitumumab in BRAF-mutant CRC. However, the small number of patients with BRAF-mutant tumors (*n* = 16), including two non-V600E-mutant tumors, limited general conclusions [43].

Interestingly, a recently published meta-analysis showed a significant benefit for triplet therapy versus doublet therapy with anti-VEGF treatment, except for BRAF-mutated mCRC [61]. Therefore, first-line treatment of this subgroup still poses an unmet medical need.

In case of successful induction therapy and disease control, capecitabine plus bevacizumab is commonly used as maintenance therapy based on results of phase III studies, regardless of mutational status of the tumor [63]. However, thanks to our increasing understanding of biological subtypes and molecular alterations, biomarker-driven studies, especially on maintenance therapy after successful induction therapy, are emerging, but positive results are still pending. The randomised FOCUS4 trial tests targeted agents in patients with advanced mCRC in molecularly stratified cohorts after 16 weeks of chemotherapy induction therapy. Patients eligible for maintenance therapy are randomised according to five molecular subtypes, i.e., BRAF mutant, PI3KCA subtype (mutation of PI3KCA gene or loss of PTEN protein), RAS mutant, RAS WT, and non-classified subtype comparing new targeted agents versus placebo maintenance or standard of care [64]. First results have recently been reported on patients with triple wild-type for *RAS*, *BRAF*, *PIK3CA* and no PTEN loss. The cohort was treated with an oral pan-HER tyrosine kinase inhibitor, AZD8931, on the background of preclinical and molecular data indicating that combined inhibition of EGFR (HER1), HER2, and HER3 might lower the risk of de-novo resistance to EGFR targeted drugs. Treatment with AZD8931 failed to show efficient anti-tumor effect, and median PFS was shorter in the AZD8931 group than in the placebo group without reaching statistical significance (2.96 months vs. 3.48 months) [65]. The MODUL trial (NCT02291289) is another biomarker-driven randomised maintenance trial. After four months of induction therapy with FOLFOX-bevacizumab, patients with stable disease or better will be separated into cohorts for maintenance therapy according to their mutational status and compared to a control arm treated with fluoropyrimidine with bevacizumab. Results from both studies are eagerly awaited in the near future and might provide a new basis for molecular-driven maintenance therapies after first-line induction.

### 5.2. Immune Checkpoint Inhibition (ICI) in mCRC

ICI has entered the field of cancer treatment in several tumor entities [66,67]. For mCRC, existing evidence revealed an impressive clinical benefit for patients with high mutational burden and mismatch repair deficiency (MMRd) [68]. MMRd CRC tumors account for approximately 5% and result in a high mutational burden, which is believed to increase the generation of neoantigens on cancer cell surface [69]. Positive results from phase II studies of ICI in previously treated MMRd mCRC led to accelerated approval of ICI by the U.S. Food and Drug Administration (FDA) in 2017, regardless of the tumor entity. Studies reported ORR of 27.9–52% and DCR of 82–90% with 1-year PFS of 34–71% and 1-year OS of 72–87% [17,18,70,71]. Very recently, interim results of the open-label, randomised phase III Keynote-177 trial were presented by Thierry André and colleagues [16]. The study investigated the efficacy and safety of pembrolizumab as first-line treatment versus standard of care (mFOLFOX6 or FOLFIRI ± anti-EGFR (cetuximab) or anti-VEGF (bevacizumab)) in MMRd mCRC. Significant improvement of PFS was detected for pembrolizumab in comparison to control arms (median 16.5 mo vs. 8.2 mo; HR 0.60; 95% CI, 0.45–0.80; *p* = 0.0002). 12- and 24-month PFS rates were 55.3% and 48.3% with pembrolizumab versus 37.3% and 18.6% with standard of care with confirmed ORR of 43.8% versus 33.1%. Importantly, grade 3–5 treatment related adverse events were 22% for pembrolizumab versus 66% for the control arm. Evaluation of OS as co-primary endpoint is pending. However, this strong clinical benefit by pembrolizumab led to approval by the FDA and approval from the EMA is currently awaited. Notably, only the presence of MMRd in mCRC has been confirmed as a predictor for ICI response in mCRC. None of the other biomarkers investigated, i.e., PD-L1 expression, mutational status in BRAF and KRAS or history of Lynch syndrome, were positive predictors [18,72].

### 5.3. Further Lines of Treatment

Sequential therapy is well established for mCRC. For further lines of therapy, targeted agents against VEGF, such as aflibercept or ramucircumab and anti-metabolite TAS-102, as well as the thyrosinkinase-inhibitor regorafenib, are approved for patients who progressed to previous treatment and regardless of mutational status of mCRC [73,74,75]. In the context of molecular driven treatments, a proof-of-concept study was provided by the phase 2 HERACLES trial, which evaluated dual-targeted treatment with the HER-2 antibody trastuzumab and the EGFR and HER-2 inhibitor lapatinib in treatment-refractory Her2-positive, KRAS exon 2-wt mCRC. Results showed ORR of 30.3%. Data on treatment safety revealed no drug-related serious adverse events. 22% of patients had grade 3 adverse events. Therefore, combination therapy seems to be safe and active in Her2-positive mCRC [19].

Patients with BRAF V600E-mutations showed a median OS of 4–6 months after failure of initial therapy, indicating the urgent clinical need in this patient cohort [76]. Interestingly, while BRAF V600E-mutated melanomas show sensitivity to BRAF-mutant inhibitor vemurafenib, efficacy in mCRC is limited [77,78,79]. Studies on resistance mechanisms suggest feedback activation of EGFR signaling, resulting in limited responses to BRAF inhibitors in CRC [79,80]. Therefore, the BEACON trial investigated combination therapy of cetuximab with a BRAF inhibitor encorafenib +/− a MEK inhibitor binimetinib versus cetuximab in combination with chemotherapy. The primary endpoints were OS and ORR in the triplet-therapy group as compared to the control group. Median OS was 9 months in the triplet-therapy group versus 5.4 months in the control group (HR for death, 0.52; 95% CI, 0.39 to 0.70; *p* < 0.001). Confirmed response rates were 26% (95% CI, 18 to 35) in the triplet-therapy group and 2% (95% CI, 0 to 7) in the control group (*p* < 0.001). Adverse events of grade 3 or higher did not differ significantly across treatment arms. Median overall survival in the doublet-therapy group was 8.4 months (hazard ratio for death vs. control, 0.60; 95% CI, 0.45 to 0.79; *p* < 0.001) [81]. These positive results led to the approval of chemotherapy-free doublet therapy for patients with progressive disease and BRAF V600E mutations by the FDA and EMA.

Besides approval for ICI for any MMRd/MSI-H tumor disease (regardless of tumor entity), larotrectinib and entrectinib gained approval for solid tumors with neurotrophic tyrosine receptor kinase (NTRK) gene fusion. Although incidence of NTRK fusion genes is low, with less than 1% in CRC, and occurs only in BRAF- and MLH1/PMS2-wt patients, testing should be performed in cases of refractory disease.

## 6. Conclusions

Multimodal and efficient treatment strategies have continuously improved outcomes of patients with CLM. Radical resection of CLM has especially greatly improved survival rates. Importantly, the benefit of perioperative or additive chemotherapy could not be confirmed in patients with favorable prognostic factors and clearly resectable CLM, regardless of underlying mutational status. However, prospective trials need to clarify specific surgical procedures or combinations of surgery with systemic treatment in patients with unfavorable clinical or molecular factors, including KRAS or BRAF mutations.

Despite increased understanding of molecular subtypes of mCRC, deciding on the best treatment approach remains challenging, especially in patients with potentially or initially unresectable CLM. The Dutch CRC group expert panel for liver metastases prospectively investigated resectability in 183 patients with CLM using a panel of radiologists and liver surgeons [82]. Interestingly, in over half of the evaluations (52%), a disagreement between experienced liver surgeons was observed. In 42 evaluations (11%), even major disagreements (resectable vs. *never*-resectable) were detected. Thus, results of this study reflect the complexity in defining resectability and highlight the importance of treatment in specialist cancer centers and decision-making supported by multidisciplinary teams. Intensive systemic therapy may render initially unresectable CLM eligible for surgical approaches with subsequent improvement of clinical outcomes. Results underline the need for reevaluation of metastatic resection in patients with initially unresectable CLM during systemic therapy. In more advanced stages, several efficient systemic therapies are available in first-line settings. However, intensity of treatments should be based on individual treatment goals considering the need for tumor mass reduction, secondary resections or disease control, as well as patient preference. Molecular and genetic diagnostic workup is crucial and strong evidence exists for the predictive and/or prognostic value of KRAS and BRAF mutations as well as for MMdr/MSI-H tumors. Generally, targeted approaches should be applied together with a chemotherapy combination. However, improved molecular stratification and identification of different biological subtypes now offers treatment approaches with chemotherapy-free regimens for selected patients, i.e., ICI monotherapy in MMDr/MSI-H mCRC and targeted combination therapy with cetuximab and encorafenib for BRAF-mutated CRC. Further advancements in molecular stratification based on large-scale gene-expression profiles have recently been provided by an international consortium. As a result of these efforts, a robust classification that defines four different subtypes could be generated: Consensus molecular subtype (CMS)1 (MSI Immune-subtype) with hypermutated, microsatellite unstable features and strong immune activation; CMS2 (Canonical-subtype) showing epithelial features, chromosomally unstable with marked WNT and MYC signalling activation; CMS3 (Metabolic-subtype) with epithelial features and evident metabolic dysregulation; and CMS4 (Mesenchymal-subtype) with prominent TGF-β activation as well as stromal invasion and angiogenesis [83]. An exploratory analysis of patients of the FIRE-3 trial that grouped 438 patients into the CRC-CMS types could show improved OS for FOLFIRI plus cetuximab over FOLFIRI plus bevacizumab for RAS-wt patients groups in CMS4 [84]. While the clinical relevance of CMS for the treatment of mCRC remains to be further defined, we now have conclusive evidence that deeper insights into tumor biology will pave the way to new and effective molecular-driven therapeutic approaches for patients with mCRC.

## Figures and Tables

**Table 1:** Resection rates and outcomes of potentially or initially unresectable CLM.

Study (Ref.)	Treatment	ORR (%)	RR (%)	mPFS (Months)	mOS (Months)
CELIM [39,40]	FOLFOX + Cet	68	38	11.2	35.8
	FOLFIRI + Cet	57	30	10.5	29
PLANET [41]	FOLFOX6 + Pan	74	34	13	37
	FOLFIRI + Pan	67	36	14	41
Ye et al. [42]	FOLFIRI/ FOLFOX6 + Cet	57.1	25.7	3-year: 10.2	3-year: 30.9
	FOLFIRI/ FOLFOX6	29.4	7.4	3-year: 5.8	3-year: 21.0
VOLFI [43]	mFOLFOXIRI + Pan	87.3	33	9.7	35.7
	FOLFOXIRI	60.6	12.1	9.7	29.8
ATOM [44]	mFOLFOX6 + Bev	68.4	56.1	11.5	30.4
	mFOLFOX6 + Cet	84.7	49.2	14.8	not reached
METHEP [45]	FOLFIRI/FOLFOX4	33	40	9.2	17.7
	FOLFIRI-HD	47	59.4	12.1	29.4
	FOLFOX7	43	43.3	8.5	26.9
	FOLFIRINOX	57	66.7	14.1	48.8
OLIVIA [46]	mFOLFOX + Bev	62	61	11.5	32.2
	FOLFOXIRI + Bev	81	49	18.6	not reached
Prodige 14 [47]	Douplet + Bev/Cet	-	45.2	-	36
	Triplet + Bev/Cet	-	56.9	-	not reached

Cet: Cetuximab; Pan: Panitumumab; ORR: overall response rate; RR: Resection rate; mPFS: median progression-free survival; mOS: median overall survival.
